# Dark Chocolate Intake Positively Modulates Gut Permeability in Elite Football Athletes: A Randomized Controlled Study

**DOI:** 10.3390/nu15194203

**Published:** 2023-09-28

**Authors:** Cristina Nocella, Elena Cavarretta, Chiara Fossati, Fabio Pigozzi, Federico Quaranta, Mariangela Peruzzi, Fabrizio De Grandis, Vincenzo Costa, Carwyn Sharp, Massimo Manara, Antonia Nigro, Vittoria Cammisotto, Valentina Castellani, Vittorio Picchio, Sebastiano Sciarretta, Giacomo Frati, Simona Bartimoccia, Alessandra D’Amico, Roberto Carnevale

**Affiliations:** 1 Department of Clinical, Internal Medicine, Anesthesiological and Cardiovascular Sciences, Sapienza University of Rome, 00161 Rome, Italy; mariangela.peruzzi@uniroma1.it (M.P.); vittoria.cammisotto@uniroma1.it (V.C.); simona.bartimoccia@uniroma1.it (S.B.); 2 Department of Medical-Surgical Sciences and Biotechnologies, Sapienza University of Rome, 40100 Latina, Italy; elena.cavarretta@uniroma1.it (E.C.); vittorio.picchio@uniroma1.it (V.P.); sebastiano.sciarretta@uniroma1.it (S.S.); fraticello@libero.it (G.F.); alessandra.damico@uniroma1.it (A.D.); roberto.carnevale@uniroma1.it (R.C.); 3 Mediterranea Cardiocentro, 80122 Napoli, Italy; 4 Department of Movement, Human and Health Sciences, University of Rome “Foro Italico”, 00135 Rome, Italy; chiara.fossati@uniroma4.it (C.F.); fabio.pigozzi@uniroma4.it (F.P.); federico.quaranta@uniroma4.it (F.Q.); 5 Villa Stuart Sport Clinic, FIFA Medical Center of Excellence, Via Trionfale 5952, 00136 Rome, Italy; fabrizio_dg@libero.it (F.D.G.); antonianigro@tiscali.it (A.N.); 6 Associazione Sportiva (A.S.) Roma Football Club, Piazzale Dino Viola 1, 00128 Rome, Italy; vincenzo.costa@asroma.it (V.C.); carwyn.sharp@asroma.it (C.S.); massimo.manara@asroma.it (M.M.); 7 Department of General Surgery and Surgical Specialty, Sapienza University of Rome, 00161 Rome, Italy; valentina.castellani@uniroma1.it; 8 IRCCS Neuromed, 86077 Pozzilli, Italy

**Keywords:** gut permeability, exercise, polyphenols

## Abstract

Gut barrier disruption can lead to enhanced intestinal permeability, which allows endotoxins, pathogens, and other proinflammatory substances to move through the intestinal barrier into circulation. Intense exercise over a prolonged period increases intestinal permeability, which can be further worsened by the increased production of reactive oxygen species (ROS) and pro-inflammatory cytokines. The aim of this study was to assess the degree of intestinal permeability in elite football players and to exploit the effect of cocoa polyphenols on intestinal permeability induced by intensive physical exercise. Biomarkers of intestinal permeability, such as circulating levels of zonulin, a modulator of tight junctions, occludin, a tight junction protein, and LPS translocation, were evaluated in 24 elite football players and 23 amateur athletes. Moreover, 24 elite football players were randomly assigned to either a dark chocolate (>85% cocoa) intake (*n* = 12) or a control group (*n* = 12) for 30 days in a randomized controlled trial. Biochemical analyses were performed at baseline and after 30 days of chocolate intake. Compared to amateur athletes, elite football players showed increased intestinal permeability as indicated by higher levels of zonulin, occludin, and LPS. After 30 days of dark chocolate intake, decreased intestinal permeability was found in elite athletes consuming dark chocolate. In the control group, no changes were observed. In vitro, polyphenol extracts significantly improved intestinal damage in the human intestinal mucosa cell line Caco-2. These results indicate that chronic supplementation with dark chocolate as a rich source of polyphenols positively modulates exercise-induced intestinal damage in elite football athletes.

## 1. Introduction

The gut barrier is a semipermeable structure that allows the absorption of essential nutrients, functions in immune sensing, and protects against toxins, food antigens, and bacteria. Indeed, the intestinal barrier is a restrictive and selective physical barrier that prevents the translocation of bacterial components such as lipopolysaccharide (LPS) into the lamina propria and then into the systemic circulation [1]. Under normal conditions, structurally, the gut barrier consists of multiple layers, from the intestinal lumen to the mucin layer comprising an inner and outer mucus layer, up to the tight and adherens junctions that keep the connections between the epithelial cells firm, preventing LPS translocation [2]. However, several pathophysiological stressors, including enteric diseases and also nutritional factors, oxidative stress, mechanical damage, antibiotics, and external stimuli, may disrupt the gut barrier inducing intestinal permeability [1]. Therefore, a healthy gut, characterized by a high degree of microbial richness, which ensures proper intestinal function, is essential for maintaining health and immunity.

Physical exercise acts as a modulator of the intestinal environment and microbiota, affecting its overall structure and functionality. However, not every type of physical exercise negatively affects the gut microbiota. Indeed, there is convincing evidence that low-to-moderate intensity exercise has positive effects on the gastrointestinal tract, including mucosa preservation, increasing microbiota diversity, and increasing butyrate-producing bacteria as well as butyrate concentrations [3]. Conversely, as early as 1992, the first data demonstrating that intestinal permeability acutely increased after marathon running was published [4]. In addition, other authors reported that aerobic exercise can induce intestinal permeability, which was affected by different exercise intensities and durations. Indeed, vigorous endurance training for ≥60 min at ≥70% of the maximum work capacity increases the intestinal permeability and is associated with a reduction in gastric motility, epithelial injury, disturbed mucosa integrity, impaired nutrient absorption, and endotoxemia with local and systemic low-grade inflammation [5].

Athletes who experienced gastrointestinal disorders showed an overall impact on health status and a reduction in performance. Recently, it has been demonstrated that in resistance-trained men, the lactulose–rhamnose (L/R) ratio and level of intestinal fatty acid-binding protein (I-FABP), both measures of intestinal cellular injury [6,7], were greater in post-exercise males than non-exercise controls [8]. In their bibliographic review, Ribeiro et al. reported the molecular and physiological changes in gut permeability caused by several types of exercise including swimming and running. These studies showed that endurance exercise can lead to an increase in intestinal permeability and that the determining factors for increased permeability are the intensity and volume of training [5].

Despite this evidence, more studies are needed to highlight the role of exercise in intestinal permeability and to identify other variables that may influence this phenomenon.

Among the several strategies that could mitigate the impact of exercise on gut permeability, nutrition and dietary supplements may play role in preventing the cell damage and gut permeability associated with exercise [9].

The impact of acute or chronic effects of a wide range of supplements, such as bovine colostrum, glutamine, probiotics, supplemental carbohydrate, and antioxidants was tested in a variety of endurance exercise protocols [10]. Despite appropriate nutrition strategies that could mitigate exercise-induced gut permeability, other supplements, such as antioxidants, demonstrated equivocal findings [11,12] and need more investigation to support any supplement additions to an athlete nutrition plan.

Dietary polyphenols constitute the biggest group of naturally occurring phytochemicals that exert a powerful antioxidant action and a wide range of pharmacological properties [13,14]. Polyphenols can also interact with the intestinal microbiota. In different murine models of metabolic disorders, polyphenols and polyphenol-rich extracts alleviated intestinal oxidative stress, and improved the inflammatory status and intestinal barrier function [15]. Moreover, polyphenols modulated the intestinal microbiota by increasing the abundance of short-chain fatty acid-producing bacteria [15] or by reducing circulating lipopolysaccharide (LPS) levels, thus improving the inflammatory status and relieving oxidative imbalances [15]. Finally, polyphenols could promote beneficial gut bacteria through their direct and collaborative effects and their inhibitory action on potential pathogenic species [9]. Specifically, in athletes, dietary polyphenol supplementation, such as with chokeberry (Aronia) [16], curcumin [17], or fruit-derived polyphenols [18], exerted beneficial effects including enhancing the redox balance and exercise performance or attenuating DOMS and muscle function deficits [16,17,18]. Few studies have reported the effect of polyphenol supplements in exercise-induced gut dysfunction [19,20], while no data about the effect of supplementation with polyphenol-rich foods on intestinal permeability in athletes are available.

Therefore, the purpose of this study was to verify the level of gut barrier permeability in elite football (soccer) players and to test if supplementation with polyphenols in the form of dark chocolate before exercise is effective at reducing the intestinal permeability.

## 2. Materials and Methods

### 2.1. Study Participants

The study was performed on 24 young elite male football players (17.2 ± 0.7 years) and 23 sex-matched amateur athletes (30.2 ± 4.6 years), as previously described [21,22]. Briefly, in this study, we compared (1) elite athletes that were all members of the Italian first-league A.S. Roma youth team (Primavera) (who train for at least 14 h/week, >6.0 METs, and had at least 8 years of competitive experience) and (2) physically active male subjects (who performed moderate-to-intense physical activity 3 days·week-1, ranging from 3.0 to 6.0 METs and/or > 6.0 METs).

Elite athletes’ enrollment, training schedules, and exclusion criteria were previously described [21]. All amateur athletes practice “mixed sports”, except for football, as already described [22] and according to the Dal Monte–Lubich sports classification [23] and the cardiovascular classification of the Olympic sport disciplines [24].

This study was approved by the institutional review board (C.E. 4662), and the randomized controlled trial was registered on ClinicalTrials.gov (Identifier: NCT03288623).

### 2.2. Study Design

The first phase consisted of a cross-sectional study performed to compare gut permeability in 24 young well-trained male elite football athletes and 23 sex-matched amateur athletes as controls by measuring the levels of LPS, zonulin, and occludin, three biomarkers that can accurately detect alterations in intestinal barrier integrity [25]. In the second phase, elite football athletes participated in a randomized controlled trial in which they were supplemented daily for 30 days with a normal diet and 40 g of commercially available dark chocolate (20 g every 12 h) vs. a normal diet to investigate the effect of polyphenols in the form of dark chocolate on markers of gut permeability.

Blood levels of LPS, zonulin, and occludin were assessed at baseline (T0) and 30 days (T30d) after the last ingestion of chocolate. During the study, the participants followed a diet adjusted according to their anthropometric and clinical characteristics and to the number of calories coming from chocolate intake while also avoiding foods with a high polyphenol content and/or additional chocolate.

### 2.3. Study Product

For the second phase, we administered tablets of the dark chocolate (cocoa solids >85%, cocoa mass, fat-reduced cocoa, cocoa butter, sugar, and vanilla). The total polyphenol content was 799 μg GAE/mL, with amounts of epicatechin and catechin of 0.65 mg/g and 0.26 mg/g, respectively.

### 2.4. Blood Sampling and Preparations

All blood samples were collected in the morning (8–9 a.m.), from the antecubital vein of fasting athletes in a seated position. Athletes were instructed to avoid intense physical training in the 24 h before the blood test. Blood was collected into the test tube (BD Vacutainer, Franklin Lakes, NJ, USA) without anticoagulant or containing trisodium citrate (3.8%, 1/10 (*v*/*v*)) to obtain serum and plasma samples, respectively. After blood centrifugation at 300× *g* for 10 min at room temperature (RT), the supernatants were separated into aliquots and kept at −80 °C until further analyses.

### 2.5. Evaluation of Gut Permeability Biomarkers

The levels of zonulin, LPS, and occludin were measured as markers of gut permeability.

The zonulin levels in serum samples and cell culture media, serum LPS levels, and plasma occludin levels were measured using commercial ELISA kits (Elabscience, Houston, TX, USA, Cusabio, Houston, TX, USA, and Novus Biologicals Centennial, CO, USA, respectively). Values are expressed as ng/mL for zonulin, pg/mL for LPS, and ng/mL for occludin.

All assays were conducted according to the manufacturer’s instructions and intra-assay and inter-assay coefficients of variation were within 10% for each assay.

### 2.6. Cell Culture

The Caco-2 cell line, a well-characterized intestinal in vitro model, was obtained from Sigma Aldrich (St. Louis, MO, USA). The cells were thawed in T75 tissue culture flasks and expanded in Caco-2/TC-7 Expansion Medium comprised of high-glucose DMEM supplemented with 1% L-glutamine and sodium pyruvate, 10% fetal bovine serum, 1% L-alanyl-L-glutamine and 1% non-essential amino acids (all reagents from Sigma Aldrich, St. Louis, MO, USA) and incubated at 37 °C in a humidified incubator with 5% CO_2_.

When the cultures reached 80% confluency, the cells were detached with 5 mL of accutase, resuspended in fresh Caco-2/TC-7 Expansion Medium, and reseeded in 60 mm plates at a density of 450 k cells/cm^2^. The passage number of the cells used in the experiments was between 2 and 6. The culture medium was replaced every 48 h.

Afterward, cells were pre-treated either with vehicle (phosphate-buffered saline, PBS) or cocoa-derived polyphenols (25–50 µg/mL) for 1 h before stimulation with lipopolysaccharides from *Escherichia coli* 0111:B4 (Sigma Aldrich, protein contamination <1%) in a range of concentrations (30–120 pg/mL) in PBS for 48 h.

To verify the mechanism, the cells were also treated with TAK-242 (1 μM in PBS; InvivoGen, Toulouse, France), a specific inhibitor of TLR4 signaling [26], before stimulation with lipopolysaccharides from *Escherichia coli* 0111:B4.

Conditioned media were collected to quantify soluble zonulin as described; pellets were analyzed by Western blot to verify barrier damage through occludin expression. The experiments were conducted on three different batches of Caco-2.

### 2.7. Extraction of Phenolic Fraction from Chocolate

Polyphenols were extracted from one gram of defatted chocolate in a total volume of 3 mL (1 × 3 mL) with 80% (*v*/*v*) acetone/water at 80 °C. Most of the polyphenols were contained in this aqueous acetone solution which was used for in vitro studies.

### 2.8. Caco-2 Cells Viability

The viability of Caco-2 cells was determined using the colorimetric MTS assay. Caco-2 cells were seeded in triplicate into 96-well microtiter plates at a density of 8000 cells/well. After being cultured in the presence of high-glucose DMEM supplemented with 1% L-glutamine and sodium pyruvate, 10% fetal bovine serum, 1% L-alanyl-L-glutamine, and 1% non-essential amino acids for 72 h, the cells were treated with LPS (0, 30, 60, and 120 pg/mL) or with a polyphenol extract (0, 25, 50, and 100 µg/mL).

The plates were incubated at 37 °C in a humidified, 5% CO_2_ incubator for 48 h. At the end of the incubation, the cell viability was determine using the CellTiter 96 AQueous Non-Radioactive Cell Proliferation Assay kit (Promega, Madison, WI, USA) following the manufacturer’s instructions.

After 48 h of treatment, the conditioned medium was removed and 20 μL MTS (3-(4,5-dimethylthiazol-2-yl)-5-(3-carboxymethoxyphenyl)-2-(4-sulfophenyl)-2H tetrazolium) was added to 100 μL of fresh culture medium and added to each well and incubated for 3 h at 37 °C in a humidified, 5% CO_2_ atmosphere. The absorbance, at a wavelength of 490 nm, was detected using a spectrophotometer (Varioskan LUX multimode microplate reader, Thermo Scientific, Waltham, MA, USA). Each test was performed in triplicate.

### 2.9. Evaluation of Oxidative Stress Biomarkers

H_2_O_2_ was measured by a colorimetric assay according to the manufacturer’s instructions (Abcam, Cambridge, UK). sNox2-dp, a measure of NOX2 activity, was evaluated with a previously reported ELISA method [27]. The values are expressed as μM for H_2_O_2_ and pg/mL for sNOX2dp. Intra- and inter-assay coefficients of variation were <10% for each assay.

### 2.10. Protein Detection, Electrophoresis, and Western Blot Analysis

The cells were scraped using ice-cold RIPA buffer in the presence of a protease and phosphatase inhibitor cocktail (10 μg/mL; Thermo Fisher Scientific, Waltham, MA, USA) and sonicated three times. Following incubation on ice for 30 min, the samples were centrifuged at 10,000× *g* for 20 min, and the supernatants were recovered and mixed with 2× Laemmli sample buffer and 2-mercaptoethanol (20%).

The Bradford colorimetric assay was used to determine the protein concentration and equal amounts of cell proteins (30 μg/lane) were separated by SDS-PAGE (10–12% polyacrylamide gel) and then electro-transferred onto nitrocellulose membranes by a Trans-Blot Turbo (Mini Nitrocellulose, Bio-Rad, Hercules, CA, USA). Incubation was performed overnight at 4 °C with primary antibodies: rabbit polyclonal anti-occludin (Sigma Aldrich) and mouse monoclonal anti-actin (Santa-Cruz Biotechnologies, Dallas, TX, USA). HRP-conjugated secondary antibodies (1:3000; Bio-Rad, CA, USA) were incubated with the membranes for 1 h. The blots were then detected using an enhanced chemiluminescence substrate (ECL Substrates, Bio-Rad, CA, USA). Densitometric analysis was performed using Image Lab software 6.1.0. The results were expressed as arbitrary units (A.U.) and represented as the mean of three independent experiments.

### 2.11. Statistical Analysis

All continuous variables were tested for normality with the Shapiro–Wilk test. Continuous variables with normal distribution are reported as mean ± standard deviation (SD), and non-parametric variables as median and interquartile range (IQR). Between-group comparisons were performed using unpaired T test for normally distributed variables and using an appropriate non-parametric test for non-normally distributed variables (Mann–Whitney U test). Correlations were calculated using the Spearman’s Rank Correlation Coefficient and described as Rs. All analyses were carried out with Prism GraphPad version 9.

## 3. Results

### 3.1. Observational Study

The clinical characteristics of the elite football players and amateur athletes were previously described [21,22] and are reported in Table 1.

**Figure 1:**
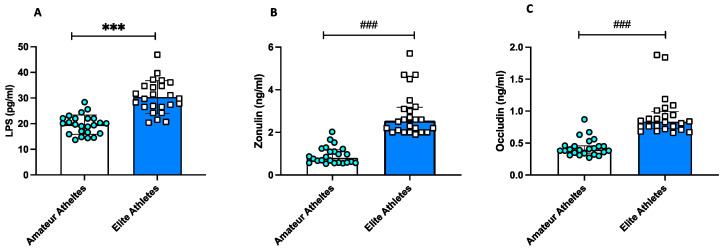
Intestinal permeability in amateurs and in elite football players. Serum levels of (**A**) LPS, (**B**) zonulin, and (**C**) occludin in amateurs (*n* = 23) and in elite football players (*n* = 24). Data are reported in the scatter dot plot and histogram as mean ± SD for Panel (**A**) or median [interquartile range] for Panels (**B**,**C**). *** *p* values < 0.001 were calculated using *t*-Test; ^###^ *p* < 0.001 using a non-parametric test (Mann–Whitney U test).

All markers of gut permeability were significantly higher in elite athletes compared to amateurs (Figure 1A–C).

**Figure 2:**
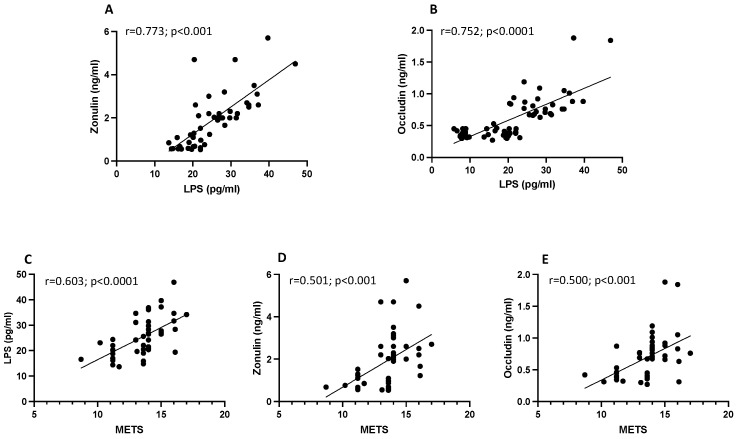
Scatter plots showing significant (2-tailed) Spearman positive correlations of zonulin (**A**) and occludin (**B**) in vertical vs. horizontal directions of LPS concentrations and of LPS (**C**), zonulin (**D**), and occludin (**E**) in vertical vs. horizontal directions of METs in 23 amateurs and 24 elite football players. Each dot represents a single individual value.

A correlation analysis showed that LPS was associated with zonulin (r = 0.773; *p* < 0.001) and occludin (r = 0.752; *p* < 0.0001) (Figure 2A,B). Interestingly, we found that the metabolic equivalents of task (METs), as reference thresholds for absolute intensities, correlated positively with LPS (r = 0.603, *p* < 0.0001), with zonulin (r = 0.501, *p* < 0.001), and with occludin (r = 0.500, *p* < 0.001) (Figure 2C–E).

### 3.2. Intervention Study

**Figure 3:**
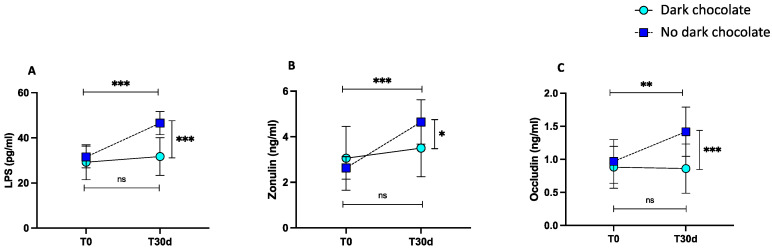
LPS (**A**), zonulin (**B**), occludin (**C**) levels before and 30 days after daily intake of 40 g of dark chocolate (green circles, continuous line) or no dark chocolate (blue square, dotted line) in elite football players. Data are presented as mean ± standard deviation. (*** *p* < 0.001; ** *p* < 0.01; * *p* < 0.05).

After 30 days of training, the control group showed increased levels of LPS compared to baseline (from 31.5 ± 4.8 pg/mL to 46.5 ± 5.15 pg/mL, *** *p* < 0.001) (Figure 3A). Moreover, we found increased levels of zonulin and occludin compared to baseline (from 2.63 ± 0.49 ng/mL to 4.65 ± 0.97 ng/mL, *** *p* < 0.001 and from 0.96 ± 1.88 ng/mL to 1.42 ± 0.37 ng/mL, ** *p* = 0.003) (Figure 3B,C). The levels of LPS, zonulin, and occludin did not change in elite football players after 30 days of dark chocolate intake compared to baseline (from 29.2 ± 7.75 pg/mL to 31.7 ± 8.37 pg/mL, *p* = 0.561, from 3.06 ± 1.40 ng/mL to 3.50 ± 1.25 ng/mL, *p* = 0.150, and from 0.88 ± 0.32 ng/mL to 0.86 ± 0.37 ng/mL, *p* = 0.99, respectively) as shown by the pairwise comparisons (Figure 3A–C). 

A significant difference between the two treatments (no dark chocolate vs. dark chocolate) was found regarding LPS (46.5 ± 5.15 pg/mL vs. 31.7 ± 8.37 pg/mL, *** *p* < 0.001) (Figure 3A), zonulin (4.65 ± 0.97 ng/mL vs. 3.50 ± 1.25 ng/mL, * *p* < 0.05) (Figure 3B), and occludin (1.42 ± 0.37 ng/mL vs. 0.86 ± 0.37 ng/mL, *** *p* < 0.001) (Figure 3C) at the end of the trial.

### 3.3. In Vitro Study

In order to substantiate the clinical effects of cocoa-derived polyphenols on gut permeability, we performed an in vitro study with the phenolic fraction extracted from dark chocolate.

**Figure 4:**
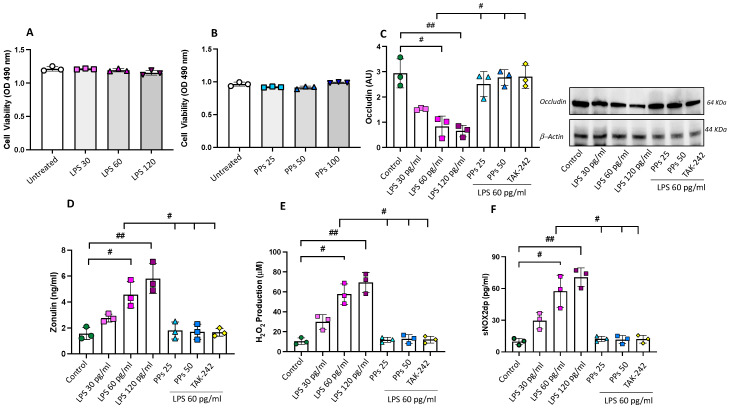
The protective effects of PPs on LPS-induced intestinal epithelial barrier damage and oxidative stress. Effects of (**A**) LPS (0–120 pg/mL) and (**B**) PPs (0–100 μg/mL) on cell viability. Caco-2 cell viability is expressed as optical density (O.D.) at 490 nm of the cells in the culture media after 48 h of incubation with each compound. Occludin expression (**C**), zonulin levels (**D**), H_2_O_2_ production (**E**), and sNOX2dp release (**F**) of Caco-2 cells stimulated with LPS (30–120 pg/mL) or with 60 pg/mL LPS in combination with cocoa-derived polyphenols (PPs, 25 and 50 μg/mL) or with TAK-242 (1 µM). ^#^ *p* < 0.05 and ^##^ *p* < 0.01 with non-parametric test.

Firstly, we investigated the effects of different concentrations of LPS (0–120 pg/mL) on epithelial cell viability to choose a non-toxic concentration. We decided to test the concentrations of LPS we found in elite athletes’ serum to verify the effects of physiologically relevant concentrations of LPS on intestinal epithelial cells. Caco-2 cells incubated with 0–120 pg/mL LPS for 48 h showed no statistical differences in cell viability, regardless of the dose of LPS, indicating that 0–120 pg/mL LPS has no cytotoxic effects on Caco-2 cells (Figure 4A).

Moreover, to determine whether polyphenol extracts (PPs) had toxic effects, Caco-2 cells were incubated with PPs (0, 25, 50, and 100 μg/mL) for 48 h at concentrations relatively similar to those found in the serum of elite football athletes after dark chocolate intake [21]. Cell viability remained unchanged in the presence of the tested doses of PPs, indicating no cytotoxic effects on Caco-2 cells (Figure 4B).

#### 3.3.1. PPs Restore LPS-Induced Intestinal Epithelial Barrier Damage

To verify the effect of LPS on epithelial barrier dysfunction, we evaluated (1) the expression of occludin, an integral membrane tight junction protein with a crucial role in the tight junction structure and permeability [28] and that, when disrupted, is released from the barriers and becomes detectable in blood [29] and (2) zonulin levels as an important modulator of intestinal permeability [30]. Therefore, Caco-2 cells were pre-treated with PPs (25 and 50 μg/mL) for 1 h, followed by LPS stimulation. The results showed that LPS, at concentrations of 60 and 120 pg/mL, significantly reduced the protein levels of occludin compared to unstimulated cells (Figure 4B). In agreement with an impairment of tight junction expression, we found increased levels of zonulin in the LPS-treated cells compared to untreated cells (Figure 4C). The treatment with polyphenol extract (25–50 μg/mL) significantly improved occludin expression (Figure 4B) and reduced zonulin levels in the cell media (Figure 4C).

Finally, pre-treatment of cells with an inhibitor of TLR4 (TAK-242) significantly reduced LPS-mediated cell damage (Figure 4C,D), confirming the role of LPS in this process.

#### 3.3.2. PPs Reduce LPS-Induced Oxidative Stress

To investigate the mechanism underlying for beneficial effect of PPs on gut damage, we measured oxidative stress by evaluating the supernatant of treated cells for (1) the concentration of soluble NOX2-derived peptide (sNOX2-dp), a measure of NOX2 activation, and (2) H_2_O_2_ production. While LPS (60 and 120 pg/mL) elicited a significant increase in oxidative stress, the pre-treatment with PPs (25 and 50 μg/mL) reduced sNOX2-dp and H_2_O_2_ levels (Figure 4E,F). The addition of a specific inhibitor of TLR4 blunted LPS-induced oxidative stress (Figure 4E,F).

## 4. Discussion

Here, we provide evidence that (1) markers of intestinal permeability are significantly increased during intensive exercise compared to moderate exercise and (2) chronic intake of dark chocolate can counteract impaired intestinal permeability induced by intense exercise during elite football players’ training sessions. In vitro, we demonstrated that polyphenols from dark chocolate can mitigate oxidative stress and epithelial permeability induced by LPS, which harms the intestinal barrier integrity.

Several pieces of evidence have described that intensive physical exercise has an impact on gut health and integrity. However, there is no detailed information regarding the appropriate exercise dose–response thresholds that do not generate adverse effects for the intestinal mucosa; it is known that the degree to which intestinal permeability is induced by acute or prolonged exercise is directly related to the duration and intensity of exercise, as well as the recovery between sessions [31].

The results of a recent meta-analysis [32] that examined the effect of an acute bout of exercise on markers of gut permeability and gut cell damage supported the current hypothesis that, in healthy participants, a single bout of exercise causes significant increases in gut damage and permeability. Moreover, experimental models suggested that intestinal barrier disruption may continue for several hours following an acute bout of exercise [33]. Furthermore, athletes engaging in intense and sustained exercise reported a gut microbiota composition that is associated with low-grade inflammatory processes [34]. Secondary to pro-inflammatory bacteria abundance, intestinal permeability parameters were also increased [34].

In our study, we found that elite athletes, participating in highly intense professional training programs, displayed increased circulating levels of zonulin and occludin, two biomarkers of impaired gut function, compared to amateur athletes engaged in moderate-to-intense exercise. Moreover, we found increased levels of serum LPS, consistent with increased gut permeability. This association was also confirmed by the positive correlation between LPS, zonulin, and occludin.

The intestinal barrier has a critical function in human health, with a role in the pathogenesis of intestinal and systemic diseases. Because of the downregulation of intestinal adhesion proteins induced by gut dysbiosis, LPS caused increases in reactive oxygen species (ROS) production [35] and translocated into the systemic circulation can elicit an inflammatory response that may contribute to atherosclerotic damage [36], clotting activation, and thrombus formation [37,38].

As athletes’ training and competition schedules increase, it is important to identify effective non-invasive strategies that are able to modulate intestinal permeability. In general, there is growing evidence that dietary supplementation with polyphenols derived from natural products can improve physical performance, with mechanisms that are most likely related to antioxidant and vascular effects. We previously demonstrated that polyphenol-rich food supplementation had a positive effect on high-intensity training elite athletes’ antioxidant status [21]. Several studies documented the effects of polyphenols in the modulation of the intestinal microbial ecosystem [39]. Only a few studies seem to have explored the potential beneficial effects of polyphenol-rich foods on intestinal permeability in humans [30] and equivocal findings on polyphenol supplementation in athletes requires further study.

In our study, we demonstrated that chronic supplementation with dark chocolate, which is a high source of polyphenols, reduces biomarkers of gut permeability induced by intense exercise compared to non-supplemented athletes.

The beneficial effects induced by polyphenols derived from dark chocolate were also confirmed in vitro, on Caco-2 cells, by analyzing the protein system that is crucial in ensuring the integrity of tight junction structures. Tight junctions have a composite molecular structure consisting of multiple protein complexes made up of several major types of proteins, including claudin, occludin, and the intracellular scaffold zonula occuldin proteins (ZO-1, ZO-2, and ZO-3). Tight junctions can be regulated by several factors, including different ROS, especially H_2_O_2_ [38], generating oxidative stress. Our data confirmed that, in vitro, LPS can increase oxidative stress and consequently destroy intestinal cell integrity. Both oxidative stress and intestinal cell injury were restored by polyphenol treatment.

Our study has limitations and implications. The fact that polyphenol supplementation modulates gut permeability in elite athletes during intensive exercise provides novel insight into a nutrition-related strategy to counteract gut injury and potentially to reduce the cardiovascular risk induced by gut-derived LPS [38].

We must acknowledge that we have not performed a direct evaluation of gut permeability. Therefore, the increased indirect biomarkers observed only indicate an intestinal injury and we only speculate that increased LPS could induce gut damage in this setting. Second, we partially evaluated the mechanisms by which polyphenols restore gut function. Previous studies indicate that polyphenols exert their biological activities in the gut at several levels. Polyphenols can modulate, through their metabolites, the intestinal microbial ecosystem, a function that could explain the reduction in LPS observed in elite athletes after supplementation. Moreover, they can downregulate inflammatory genes, such as NF-κB, and cytokine production and up-regulate antioxidant genes [40]. However, the mechanisms involving the gut microbiota and permeability modulation remain unclear and further mechanistic studies are necessary. As we only included male and mixed sport athletes to enhance comparability among the groups, our results cannot be applied to all athletes, as skill, endurance, and power athletes, but specific studies should be carried out in those populations as well as in female athletes. Finally, we must acknowledge that there is a significant difference in the average age among amateur athletes and elite football players. However, previous data showed that the functional capacity of the intestinal barrier is maintained despite age [41,42].

## 5. Conclusions

Our results highlight that highly intense exercise impaired gut permeability and that polyphenols from dark chocolate are active natural products with excellent antioxidant and anti-inflammatory properties that effectively protected intestinal cells against LPS-induced injury. These results suggest that polyphenol-rich foods such as dark chocolate may be effective adjuvants for preventing LPS-associated intestinal damage in football athletes.

## Figures and Tables

**Table 1:** Baseline characteristics of amateur athletes and elite football players.

	Amateur Athletes (*n* = 23)	Elite Football Players (*n* = 24)	*p*
Age (years)	30.2 ± 4.6	17.2 ± 0.7	<0.0001
Gender (M/F)	23/0	24/0	-
WBCs (×10^3^ μL)	7.2 ± 2.2	5.6 ± 1.3	<0.01
PLTs (×10^3^ μL)	215.3 ± 40.3	228.0 ± 39.7	0.282
RBCs (×10^6^ μL)	5.1 ± 0.4	5.8 ± 0.3	<0.0001
Cholesterol (mg/dL)	185.1 ± 30.8	172.3 ± 29.4	0.151
Glycemia (mg/dL)	89.0 ± 28.8	83.5 ± 15.2	0.414
BMI	24.3 ± 1.9	22.5 ± 1.5	<0.001
Heart rate at rest (bpm)	62.1 ± 10.4	56.3 ± 11.6	0.08
Systolic blood pressure (mmHg)	114.8 ± 11.2	111.7 ± 7.9	0.284
Diastolic blood pressure (mmHg)	74.1 ± 7.2	70.9 ± 6.8	0.128
Training per week (h)	5.1 ± 2.0	18.0 ± 2.0	<0.0001
Maximum workload (METs)	12.3 ± 1.9	15.1 ± 1.4	<0.001
Peak heart rate (bpm)	164.7 ± 6.9	169 ± 11.5	0.131

WBC: white blood cell; PLT: platelet; RBC: red blood cell; BMI: body mass index. Data are expressed as mean values ± standard deviation (SD).

## Data Availability

The data presented in this study are available on request from the corresponding author.
